# Antibody Therapy for Pediatric Leukemia

**DOI:** 10.3389/fonc.2014.00082

**Published:** 2014-04-21

**Authors:** Aditi Vedi, David S. Ziegler

**Affiliations:** ^1^Kids Cancer Centre, Sydney Children’s Hospital, Randwick, NSW, Australia; ^2^School of Women and Children’s Health, University of New South Wales, Randwick, NSW, Australia

**Keywords:** antibodies, monoclonal, bispecific antibodies, conjugated antibodies, childhood leukemia, acute lymphoblastic leukemia, acute myeloid leukemia

## Abstract

Despite increasing cure rates for pediatric leukemia, relapsed disease still carries a poor prognosis with significant morbidity and mortality. Novel targeted therapies are currently being investigated in an attempt to reduce adverse events and improve survival outcomes. Antibody therapies represent a form of targeted therapy that offers a new treatment paradigm. Monoclonal antibodies are active in pediatric acute lymphoblastic leukemia (ALL) and are currently in Phase III trials. Antibody-drug conjugates (ADCs) are the next generation of antibodies where a highly potent cytotoxic agent is bound to an antibody by a linker, resulting in selective targeting of leukemia cells. ADCs are currently being tested in clinical trials for pediatric acute myeloid leukemia and ALL. Bispecific T cell engager (BiTE) antibodies are a construct whereby each antibody contains two binding sites, with one designed to engage the patient’s own immune system and the other to target malignant cells. BiTE antibodies show great promise as a novel and effective therapy for childhood leukemia. This review will outline recent developments in targeted agents for pediatric leukemia including monoclonal antibodies, ADCs, and BiTE antibodies.

## Introduction

Leukemia is the most common pediatric malignancy and is still the most frequent cause of death of all childhood malignancies (1). Despite significant progress in cure rates since the 1970s, relapsed and refractory acute lymphoblastic leukemia (ALL) still results in a high burden of disease (2) with a 5-year survival of ~30%. Moreover, acute and long-term adverse effects of systemic conventional chemotherapy and radiotherapy limit quality of life for survivors (3). Acute myeloid leukemia (AML) is less common than ALL in the pediatric population and carries a poorer prognosis. While 80% of children with newly diagnosed AML will achieve remission, the overall cure rate remains unchanged at 50–60% (4).

Over the past decade, leukemia outcomes have improved as a result of optimizing chemotherapy; tailoring treatment to individual patients, for example, by monitoring for minimal residual disease (MRD); and utilizing more sophisticated hematopoietic stem cell transplantation (HSCT) techniques. However, current conventional cytotoxic drugs have limitations including their narrow therapeutic window. This leads to systemic cytotoxicity, due to non-selective mechanisms of action that affect both normal and neoplastic cells (5, 6). Thus, novel therapeutic approaches are needed to overcome these limitations, reduce adverse effects, and improve disease-free and overall survival (OS), especially in patients with relapsed or refractory disease. Targeted therapies that deliver drugs specifically to malignant cells while minimizing exposure to normal tissues represent one therapeutic approach.

Since the role of the immune system in the recognition and elimination of malignant cells has been better understood (7), monoclonal antibodies and antibody-drug conjugates (ADCs) have been explored and developed as potential novel therapies for both hematological and solid tumors. Malignant cells, such as leukemic blasts, express antigens on their surface that can be selectively targeted by monoclonal antibodies. This minimizes generalized side effects and allows directed delivery of highly potent drugs. They have longer circulating half-lives, greater accumulation in tumor cells, and fewer systemic side effects than traditional chemotherapeutic agents (8).

Leukemic cells are particularly well suited to these novel antibody based treatment strategies since their surface antigen markers are well characterized, readily accessible in the circulation and shared almost exclusively with precursor cells in the hematopoietic system, the depletion of which can be transiently tolerated (9).

One of the factors that contribute to the efficacy of antibody based therapies includes the level of expression of the target antigen. Antigen targets are ideally expressed in high concentrations on malignant cell surfaces but not on normal cells, thus enhancing the selectivity and minimizing the systemic side effects of the drug. Rituximab is a naked monoclonal antibody against CD20 that is effective in non-Hodgkin lymphoma (NHL) where 100,000 antigens are present on the surface of each cell (10). However, high level antigen expression is not a prerequisite for clinical benefit, especially when treated with ADCs. For example, AML cells express ~5000–10,000 copies of CD33 on each cell’s surface, which is sufficient to produce sensitivity to gemtuzumab ozogamicin (GO) (11). Antigen expression on non-vital organ or cell populations may be acceptable, such as CD19, CD20, and CD33, which are markers for B cells and myeloid cells. Most patients can temporarily tolerate the elimination of these cells.

The selection of “functional antigens” that are essential for cell survival is advantageous to prevent the malignant cells becoming resistant by down-regulating the specific target antigen. The target antigen should be expressed in all or most patients with the disease, or at least be measurable through flow cytometry to allow selection of patients likely to benefit. It is ideally expressed throughout the disease course (12).

Rapid internalization of the bound antigen-ADC complex is desirable, a process that usually occurs through receptor mediated endocytosis. The catabolic environment within lysosomes then provides ideal conditions for effective drug release (13). In contrast, slow internalization is preferred for naked monoclonal antibodies to allow them to trigger antibody-dependent cellular cytotoxicity (ADCC) or complement-dependent cytotoxicity (CDC) upon binding to the target antigen (14).

Unconjugated monoclonal antibodies are the archetype on which targeted therapies are modeled and rituximab has demonstrated high levels of therapeutic utility within this class of drugs (15). Murine antibodies (derived entirely from mice) or chimeric antibodies (constructed from variable regions derived from a murine source and constant regions from a human source) result in an immune response and the generation of human anti-murine antibodies that can limit their efficacy and lead to resistance. Humanized and entirely human antibodies circumvent this limitation as their constant and variable regions are human-derived and hence less likely to generate antibodies. They have half-lives of days to weeks in the human circulation (15).

Antibody-drug conjugates combine the specificity of monoclonal antibodies with the potency of highly effective cytotoxic drugs that cannot be delivered systemically. After binding to the target antigen, the ADC-antigen complex is internalized and transported to intracellular organelles, where the cytotoxic drug is released and causes cell death.

Bispecific antibodies have been recently developed and use a similar approach, but rather than enhancing the selectivity of a chemotherapeutic compound, they seek to engage the patient’s own immune system to target the tumor cell. They typically contain two binding sites – one that targets an antigen on the cancer cell and one that targets immune cells, such as T cells, and thus engages them to attack the malignant cell.

Other conjugated immunotherapies currently under investigation for the treatment of leukemia include pretargeted radioimmunotherapy, which combines antibodies with α, β, or γ-emitting radionuclides (16). These immunoconjugates are beyond the scope of this review.

The current state of clinical development of antibodies for treatment of pediatric leukemia are summarized in Table 1 and discussed in detail below.

**Table 1:** **Clinical development of antibody therapy for pediatric leukemia**.

Drug	Target; indication	Antibody	Cytotoxic	Linker	Clinical phase	Pediatric trial phase	Response rate
**UNCONJUGATED MONOCLONAL ANTIBODIES**							
Rituximab	CD20; NHL, ALL	Chimeric	–	–	III	III	89% OS in ALL (18)
Epratuzumab	CD22; ALL	Humanized	–	–	III	III	52% CRR in ALL (79)
Ofatumumab	CD20; ALL, NHL	Humanized	–	–	II	–	90–100% ORR in NHL (21)
Alemtuzumab	CD52; CLL	Humanized	–	–	III	II	8% ORR in ALL (22)
**ANTIBODY DRUG CONJUGATES**							
Gemtuzumab ozogamicin	CD33; AML	Humanized	Calicheamicin	Disulfide linker	III	III	25–30% ORR in AML (35)
Inotuzumab ozogamicin (CMC-544)	CD22; ALL, DLBCL	Human	Calicheamicin	Acid labile linker	II/III	I	57% ORR in ALL (53)
SAR3419	CD19; DLBCL, ALL, HL	Humanized	DM4	Disulfide linker	II	Preclinical	N/A
SGN-CD33A	CD33; AML	Humanized	Calicheamicin	Protease-cleavable linker	I	–	N/A
AVE9633	CD33; AML	Humanized	Maytansinoid (DM4)	Disulfide linker	I	I	N/A
SGN-CD19A	CD19; B cell ALL	Humanized	MMAF		I	I	N/A
DCDT2980S	CD22; NHL	Humanized	MMAE	Protease-cleavable linker	0	–	N/A
**BISPECIFIC ANTIBODIES**							
Blinatumomab	CD19/CD3ε; B cell ALL, DLBCL	Humanized	–	–	II	I/II	41% ORR in ALL (73)

*ALL, acute lymphoblastic leukemia; AML, acute myeloid leukemia; NHL, non-Hodgkin’s lymphoma; HL, Hodgkin lymphoma; DLBCL, diffuse large B cell lymphoma; ALCL, anaplastic large cell lymphoma; CLL, chronic lymphocytic leukemia; MMAE, monomethyl auristatin E; MMAF, monomethyl auristatin F; OS, overall survival; ORR, overall response rate; CRR, complete remission rate*.

## Unconjugated Monoclonal Antibodies

Unconjugated monoclonal antibodies selectively target antigens expressed on malignant cells and cause cell killing by three main mechanisms: ADCC through the engagement of NK cells, macrophages, and neutrophils; antibody-dependent cellular phagocytosis (ADCP); and CDC (12). They have generally shorter circulating half-lives than ADCs and the risk of antibody development against murine proteins can limit their utility (14). The majority of naked monoclonal antibodies are used in combination with chemotherapy as they have insufficient cytotoxic activity when delivered as monotherapy.

### Anti-CD20 antibodies

Rituximab, a chimeric anti-CD20 monoclonal antibody, is one of the earliest examples of this class and is now used as standard front-line therapy in adult NHL and second line treatment in other hematologic malignancies (17, 18). CD20 expression is found in >95% of B cell lymphomas, particularly in mature cell malignancies such as Burkitt lymphoma, making this an ideal target. Anti-CD20 antibodies have an effect on cell signaling and induce apoptosis through ADCC and CDC mechanisms. Secondary to CD20+ B cell depletion from the blood, marrow, and lymph nodes, rituximab is associated with hypogammaglobulinemia and increased risk of infections, particularly some viral infections including cytomegalovirus and hepatitis B (16).

Rituximab has been investigated in children primarily as a therapy for Burkitt (mature B cell) leukemia/lymphoma. Its activity has been demonstrated as a single agent (19) and the Children’s Oncology Group (COG) recently completed a single arm Phase II trial of rituximab in combination with standard chemotherapy in pediatric patients with newly diagnosed mature B cell leukemia and/or lymphoma, with results pending (NCT00057811). An international Phase III randomized trial is currently evaluating the benefit of the addition of rituximab to standard therapy in children with newly diagnosed mature B cell leukemia/lymphoma (NCT01516580).

CD20 is expressed in ~50% of pre-B ALL with increased expression in childhood ALL observed after induction chemotherapy. The role of anti-CD20 monoclonal antibody therapy in this condition is yet to be defined. Rituximab resistance is emerging as a clinical issue in adult leukemia and lymphoma, leading to the development of newer generation anti-CD20 antibodies including ofatumumab, ocrelizumab, and veltuzumab (20).

Ofatumumab, a fully humanized anti-CD20 monoclonal antibody, is currently undergoing a Phase II study in combination with the hyper-CVAD regimen (cyclophosphamide, vincristine, adriamycin, and dexamethasone) as first line treatment for adult patients with CD20 positive ALL. A Phase II trial of ofatumumab in combination with conventional chemotherapy (cyclophosphamide, doxorubicin, vincristine, and prednisone; O-CHOP) in patients with follicular lymphoma resulted in a 90–100% overall response rate (21).

### Alemtuzumab

Alemtuzumab (Campath) is a humanized anti-CD52 monoclonal antibody that is used predominantly for the treatment of refractory chronic lymphocytic leukemia (CLL) in adult populations and for graft versus host disease prevention in pediatric HSCT recipients. CD52 is broadly expressed across all normal T and B cell lymphocytes, except plasma cells, as well as on some myeloid cells. Due to this broad expression of CD52 antigen, the major toxicity is immunosuppression and opportunistic infections. CD52 is also highly expressed across a variety of malignant cells including in childhood precursor B cell ALL. It was therefore evaluated by the COG as a single agent in a Phase II study for children with relapsed or refractory childhood ALL. Limited activity was seen with a response rate of only 8%, suggesting no defined role in this disease, although poor accrual led to early termination of the trial and a small sample size of nine fully evaluable patients (22).

### Epratuzumab

Epratuzumab, is a humanized anti-CD22 monoclonal antibody that exerts its anti-cancer efficacy through ADCC activity and biologic activity through B cell receptor modulation. CD22 expression is restricted to B cells including immature and mature B cells, but not pro-B or plasma cells, and is expressed on the majority of pre-B ALL cells, making it an attractive target for immunotherapy. Epratuzumab was initially studied for the treatment of lupus and is currently in Phase III stages of development for this indication. It has shown promising results in adult NHL and diffuse large B cell lymphoma (DLBCL) in combination with rituximab and standard chemotherapy (23).

Epratuzumab represents the monoclonal antibody that is most progressed in the treatment of childhood pre-B ALL. A pilot COG study showed that it was well tolerated in 15 children with relapsed ALL with toxicity limited to mild infusion related reactions. During a single agent treatment window limited activity was seen. However, of the 12 patients who received antibody in combination with chemotherapy, 9 achieved a complete remission (CR), and in 7 their MRD became undetectable (24). In a larger, follow up, Phase II study, 114 children received epratuzumab in combination with re-induction chemotherapy for relapsed ALL. While remission rates did not differ from historical controls, those who obtained CR had significantly lower MRD levels than patients in previous reports treated with the same chemotherapy regimen (25). These results suggest that treatment with epratuzumab may improve the quality of remission in relapsed patients and hence overall outcomes. This hypothesis is currently being studied in a large, randomized, international Phase III relapsed pediatric ALL study. As such, epratuzumab will be the first monoclonal antibody to be evaluated in a randomized Phase III setting for childhood pre-B ALL.

## Conjugated Monoclonal Antibodies

Antibody-drug conjugates have been shown to be more active than unconjugated monoclonal antibodies targeting the same surface antigen in preclinical studies. For example, SAR3419, an ADC comprised of the anti-CD19 antibody huB4 and the maytansine derivative DM4, has greater anti-tumor activity compared with the monoclonal antibody huB4, the drug conjugate DM4 alone or the unconjugated anti-CD20 rituximab (26). Due to limitations in binding sites on each antibody, only a small amount of active drug can be expected to be delivered to target cells, a barrier that can be overcome by utilizing highly potent cytotoxic agents (27).

Leukemic cells are prime targets for ADCs as they express several human antigens that are not commonly expressed on normal cells and are easily accessible in the circulation (11, 27). Additionally, anti-drug antibody formation, and hence chemotherapy resistance, is reduced in leukemia due to the commonly associated immunosuppression and depletion of B cells and thus decreased ability to form antibodies. Since they do not require active immunological responses to exert their clinical activity, they may also be effective in profoundly immunocompromised patients. The ADC target antigens in leukemia are well characterized, and their expression on normal cells (such as precursor B cells and myeloid cells) can be tolerated due to the sophisticated supportive care available in clinical practice, and the ability of these cells to regenerate (9).

The target antigen, potency of the cytotoxic agent and stability of the linker that joins the two elements of the ADC have interdependent effects on the properties of the drugs. They determine the clinical activity and tolerability of the ADC (28).

### Linkers

Linker technology is an area in ADC drug development that has progressed rapidly in recent years. There are four main types of linkers currently in use (27):

1. Acid labile-hydrazone linkers that are degraded in the acidic environment of lysosomes (pH ~5) (Unstable, acid-cleavable).
2. Disulfide-based linkers that are selectively cleaved in the intracellular milieu of the cytosol (Unstable, acid-cleavable).
3. Peptide linkers such as citrulline–valine that are highly stable in circulation and are degraded by lysosome proteases in the target cells. They are generally more stable than disulfides or hydrazones.
4. Non-cleavable thioether linkers that release the active cytotoxic drug after degradation of the antibody in the lysosomes.

Stable linkers such as peptide linkers have the advantage of extending the half-lives of cytotoxic drugs from hours to several days. Unstable linkers can reduce the half-lives of monoclonal antibodies, resulting in free antibody, which will competitively bind to the target antigen thereby reducing the efficacy of the ADC (29). Intermediate linker stability results in the most effective ADCs, since highly stable linkers result in decreased cytotoxic drug release following internalization of the ADC-antigen compound. For example, a serum-stable but intracellularly cleavable linker used to join epratuzumab to SN-38 used in B cell malignancies was shown to be 40- to 55-fold less potent than the more labile CL2A linker in *in vitro* studies (30).

Antibody-drug conjugates linked by a disulfide linker (but not thioether bond) are capable of exerting a bystander effect on cells that express none or low levels of the target antigen. Hence ADCs can be engineered to exert a bystander effect by being linked by a disulfide bond or exert more precise killing of cells expressing the target antigen and sparing nearby normal cells by being linked by a thioether bond. The bystander effect may be beneficial particularly in solid tumors whereby damaging supporting structures including endothelial cells, neovasculature, and stromal cells can enhance the efficacy of the ADC (31). However, in leukemia precision of the ADC in targeting circulating malignant cells is more desirable.

There is still an ongoing effort to further improve linker technology to improve the efficacy and reduce toxicity of ADCs. Newer linker technologies include flexible polymer linkers (Mersana Therapeutics), which allow greater drug loading (15–20 drugs per antibody), as well as the use of antibody fragments. This allows the use of less potent cytotoxics and hence potentially reduces generalized toxicity (32).

### Cytotoxic drug

Historically ADCs combined monoclonal antibodies with standard chemotherapeutic agents including anthracyclines (doxorubicin), methotrexate, and vinca-alkaloids (vinblastine) due to their availability and known cytotoxic properties. More recently highly potent cytotoxic drugs have been used that cannot be delivered systemically without being conjugated to specific antibodies via a stable linker.

Auristatins and maytansines exert their cytotoxic activity through inhibition of microtubule assembly by binding to tubulin at the same site as vinca-alkaloids. These agents are 50- to 200-fold more potent then vinca-alkaloids and they cause G_2_/M phase cell cycle arrest and apoptotic cell death. Calicheamicin is an enediyne antibiotic and DNA strand cleaving agent that causes double-strand breaks, leading to cell apoptosis. Each is 100- to 1000-fold more potent than conventional chemotherapy drugs, but has little to no cytotoxic activity at the maximum tolerated dose achievable in the clinic if used alone.

Pharmacokinetic studies have shown that an average of four drugs per antibody binding site produces a stable compound that effectively delivers optimal drug concentrations into malignant cells that express the target antigens (33, 34). More heavily loaded drug concentrations tend to be rapidly cleared from the circulation or cause aggregation and impair antigen binding (12). Less loaded conjugates result in free monoclonal antibody, which competitively binds to the target antigen, resulting in a shorter half-life (13).

## CD33

CD33 is an antigen expressed in significant levels by 90% of leukemic blasts in AML and immature normal cells of the myelomonocytic lineage but that is absent from normal hematopoietic stem cells (12), making it an optimal target.

### Gemtuzumab ozogamicin

Gemtuzumab ozogamicin (Mylotarg) is the first example in the ADC class of drugs to receive FDA approval. It was approved in 2000 for the treatment of AML after undergoing trials as both monotherapy and combination therapy with standard treatment in adult AML patients (35). It is a humanized anti-CD33 monoclonal antibody linked to calicheamicin. The antibody is linked to the cytotoxic drug via an acid labile disulfide linker, which is hydrolyzed within the acidic environment of lysosomes and endosomes in target cells to release calicheamicin as an active drug.

Conflicting results have been seen in adult AML patients treated with GO. As monotherapy in patients >60 years with relapsed CD33 positive AML, GO resulted in an overall response rate of 25–30%. However, GO was withdrawn from the market by its manufacturer in June 2010 due to the results of a Phase III randomized controlled trial that showed no additional benefit of GO in combination with standard therapy (daunorubicin and cytarabine) over standard therapy alone for adult AML (36). Additionally, fatal toxicity secondary to veno-occlusive disease (VOD) was reported in the GO arm, with an incidence of ~2% and increased risk post HSCT (37). GO’s efficacy was thought to be limited by heterogeneous drug conjugation, linker instability, and a high incidence of multi-drug resistance (38).

Gemtuzumab ozogamicin has been studied extensively in pediatric AML. In a Phase I pediatric study it induced remission as a single agent in 28% of patients with relapsed or refractory CD33 positive AML. The main adverse events reported were marrow suppression and VOD. The latter occurred in 40% of patients who underwent HSCT after GO, and one patient prior to HSCT. This patient subsequently underwent HSCT without developing VOD (4). In a randomized Phase III study of GO as post-consolidation therapy for children with AML, the drug was well tolerated, however no survival benefit was seen (39). GO was recently evaluated in a randomized Phase III COG trial in pediatric patients newly diagnosed with AML. The addition of GO to standard chemotherapy did show improved overall event-free survival and relapse-free survival but with no significant difference in OS (40, 41).

A low fractionated dose regimen of GO as first line therapy in adults with previously untreated AML showed significant improvement in event-free, relapse-free, and OS compared with standard chemotherapy, and was generally well tolerated apart from hematological toxicity (thrombocytopenia) in a Phase III randomized open-label trial (ALFA-0701) (42). This suggests that this regimen may allow for the delivery of higher cumulative doses and improve outcomes.

Several case reports have identified the potential of GO monotherapy in inducing remission in relapsed CD33 positive ALL, which represents 15% of pediatric and adult ALL (43–47).

### SGN-CD33A

SGN-CD33A is a humanized anti-CD33 antibody conjugated to a highly potent, synthetic DNA cross-linking pyrrolobenzodiazepine dimer via a protease-cleavable linker. It causes DNA damage with cell cycle arrest and apoptotic cell death to exert its efficacy in CD33 positive AML (38). A Phase I dose finding study is currently recruiting adult patients with CD33 positive AML (NCT01902329).

### AVE9633

This humanized antibody (huMy9-6) that targets CD33 is linked to the maytansinoid (DM4) via a disulfide linker. It is currently undergoing a Phase I trial in relapsed or refractory CD33 positive AML in adults (NCT00543972). Results are pending, but one complete response and one partial response was observed from the first 17 patients enrolled in the study (12).

## CD22

As discussed above, CD22 has been identified as an ideal target for ADCs due to high expression on the surface of malignant B-lineage leukemia (>90% of B cell ALL) and lymphoma cells and rapid internalization after binding (48, 49). Since the CD22 antigen undergoes constitutive endocytosis, it is well suited for intracellular drug delivery.

A number of CD22 targeted ADCs and recombinant immunotoxins are currently in development for pediatric B-lineage ALL and NHL, as well as adult hairy cell leukemia (49–52).

### Inotuzumab ozogamicin

Inotuzumab ozogamicin (IO, CMC-544) is a human anti-CD22 monoclonal antibody linked to calicheamicin, which was shown to induce CR in 39% of adults and children with relapsed and refractory ALL with an overall response rate of 57% in a Phase II trial (53). A Phase I/II dose escalation trial in adults resulted in 71 and 88% OS in DLBCL and follicular lymphoma, respectively (54). It is currently undergoing a randomized Phase III trial in adults with a pediatric ALL Phase I trial planned. Despite lower CD22 expression on ALL cells compared to lymphoma cells, IO had similar cytotoxicity against both types of cells in preclinical *in vitro* studies (55).

### DCDT2980S

DCDT2980S is a humanized anti-CD22 antibody linked to the potent monomethyl auristatin E (MMAE) cytotoxic agent via a protease-cleavable linker, and is capable of inducing complete tumor regression in xenograft models of NHL (56).

## CD19

CD19 is a transmembrane glycoprotein and a pan-B cell marker expressed throughout B cell development with the exception of mature plasma cells. It has threefold higher expression in mature B cells compared with immature B cells (57) and is one of the earliest B cell restricted antigens. It plays an important role in maintaining balance between immunity and autoimmunity. CD19 is integral to B cell differentiation through receptor signaling at multiple stages of B cell development, which allows anti-CD19 antibodies to target various B cell malignancies including immature precursor B cells in ALL (58). CD19 is almost universally expressed in all pediatric ALL blast cells (59), which has led to interest in the development of CD19 targeted antibodies for the treatment of ALL.

### SAR3419

SAR3419 is comprised of a humanized monoclonal IgG antibody targeting CD19 (huB4) and a maytansine derivative and highly potent cytotoxic drug (DM4) conjugated via a cleavable disulfide cross-linking agent (*N*-succinimidyl-4-2-pyridildithio butanoic acid, SPDB). It has a half-life of 4–6 days *in vivo*. The humanized B4 monoclonal antibody alone directed against CD19 was not found to have any *in vivo* activity against a variety of lymphomas in mouse models (26).

A Phase I trial in adults with relapsed or refractory CD19 positive B cell NHL resulted in a 33% objective response rate with further results pending (60). Another Phase I first-in-man clinical trial in patients with relapsed lymphoma demonstrated a reduction in tumor size in 47% of adult patients (61). The main dose-limiting toxicity in Phase I trials has been reversible corneal microcystic epithelial changes. There has been a notable lack of significant hematological toxicities (58).

SAR3419 was identified through the National Cancer Institute’s Pediatric Preclinical Testing Program as a potentially highly effective therapy for pediatric ALL. Further preclinical studies suggest that SAR3419 is highly effective in combination with standard induction chemotherapy (vincristine, dexamethasone, and l-asparaginase) for CD19 positive ALL, including chemoresistant subtypes such as Philadelphia positive ALL and infant MLL-ALL. SAR3419 induced durable remissions in highly chemoresistant ALL xenografts and effectively prevented relapse in hematolymphoid and peripheral organs (except the CNS) when administered in combination with standard chemotherapy (62). A Phase I/II trial in adult ALL patients is currently recruiting, and a pediatric Phase I trial is currently planned.

### SGN-CD19A

This humanized anti-CD19 monoclonal antibody conjugated to the auristatin derivative monomethyl auristatin F (MMAF) showed positive results in a first-in-human Phase I trial of patients with relapsed or refractory B cell ALL and lymphoma, including pediatric patients. Of a total eight patients with leukemia, one achieved CR and four experienced clinical improvement. The main reported adverse effects were headache, fever, nausea, fatigue, and blurred vision (63).

## Other Antibody-Drug Conjugates

Antibody fusion proteins combine the cytotoxic portion of a protein toxin produced by bacteria, fungi, or plants, and a monoclonal antibody directed at antigens expressed on malignant cell surfaces. These cytotoxic agents inhibit protein synthesis and induce apoptosis. Moxetumomab pasudotox is an example of this class of drugs that is composed of a humanized anti-CD22 monoclonal antibody and a 38 kDa fragment of the pseudomonas exotoxin A called PE38 (64). A Phase I trial of moxetumomab pasudotox resulted in 3 CRs out of 12 pediatric patients with pre-B cell ALL (65) and a Phase II study of pediatric patients with relapsed or refractory B cell ALL or NHL is currently planned.

## Bispecific Antibodies

Since the recognition of tumor immune surveillance and the role of T cells in this process (66, 67), various T cell based therapeutic approaches have been developed to control cancer growth or induce tumor regression. These include anti-cancer vaccines, T cell activating antibodies and adoptive transfer of autologous *ex vivo* expanded T cells (68). Most of these strategies are subject to tumor escape mechanisms through down-regulation of surface antigens and loss of molecules involved in T cell recognition. Additionally, conventional antibodies cannot recruit T cells as they lack an Fcγ receptor.

Bispecific T cell engager (BiTE) antibodies can largely overcome these limitations by directly engaging and recruiting pre-existing, antigen-experienced, polyclonal T cells at the invariant CD3 receptor as well as antigens on malignant cell surfaces and bringing them into close proximity. This then triggers the signaling cascade of the T cell receptor complex and redirects endogenous T cells against specifically targeted malignant cells. Granules containing granzymes and perforin fuse with the T cell membrane and discharge their cytotoxic contents. (69). This local T cell activation has the potential to be used to monitor the efficacy of BiTE antibody-drugs.

Bispecific T cell engager antibodies are rapidly emerging as an exciting novel targeted cancer therapy, particularly in hematological malignancies. The therapeutic mechanism of BiTE antibodies is relatively resistant to immune escape mechanisms as they utilize the patient’s own immune system for their efficacy. Since these drugs rely on functional immune effector cells for activity, challenges exist around their administration in conjunction with myelosuppressive chemotherapy. While a precise role is yet to be defined, their administration after allogeneic HSCT may be effective.

### Blinatumomab

Blinatumomab is an anti-CD19/anti-CD3ε bispecific antibody in clinical development for the treatment of B-lineage hematologic malignancies. It is designed to transiently engage primed cytotoxic effector memory T-lymphocytes for targeted killing of malignant B cells, which uniformly express CD19 (70).

There have been very promising results emerging from Phase II trials with blinatumomab, indicating that it is a highly efficacious anti-leukemia drug. In a recent long-term analysis of a Phase II trial with a median follow up of 33 months, blinatumomab induced an 80% MRD response rate in adults (16 of 20 patients) with B cell ALL and persistent or relapsed MRD (71). Blinatumomab has been reported to induce CR in three cases of pediatric patients with relapsed and refractory B cell ALL after allogeneic HSCT (72). A pediatric Phase I/II trial of blinatumomab in children with relapsed or refractory B cell ALL resulted in an overall response rate of 41% with a 32% CR rate (73). The most significant reported adverse events are reversible central nervous system toxicities including encephalopathy, tremor, and aphasia (20).

An adult Phase II trial in patients with refractory or relapsed DLBCL is also currently recruiting patients (NCT01741792) and a number of other Phase II trials with blinatumomab are planned including patients with Philadelphia positive B cell ALL (NCT02000427) and MRD positive ALL (NCT00560794). A Phase III trial of blinatumomab for patients with refractory or relapsed B cell ALL is also planned (NCT02013167).

Blinatumomab does have a relatively short half-life of 2–3 h due to rapid renal clearance, making continuous infusion over 4–8 weeks via portable mini-pump the optimal mode of delivery (69), which presents challenges in the pediatric population. The dependence on circulating immune cells also limits the ability to combine the treatment with standard cytotoxic and myelosuppressive therapies. However the striking efficacy and has generated great interest with a potential future role in the management of MRD positive disease.

## Chimeric Antigen Receptors

Although a detailed discussion is beyond the scope of this review, chimeric antigen receptors (CARs) are also emerging as effective therapies for hematological malignancies. CARs are T cells genetically modified and linked to an antibody directed against malignant cell surface antigens. They exert their cytotoxicity through T cell mediated signaling pathways. CARs directed against CD19 antigens have been effective in hematological malignancies with an overall CR rate of 88% in adults with refractory or relapsed B cell leukemia (74). A number of ongoing clinical trials using CARs directed against CD19 have demonstrated efficacy in pediatric patients with B cell leukemia and lymphoma (75). Grupp et al. reported CRs in two pediatric patients with relapsed and refractory B cell ALL with CTL019 CAR T cells. The most significant dose-limiting toxicities were the cytokine-release syndrome, requiring cytokine blockade with etanercept and tocilizumab, and B cell aplasia (76).

## Conclusion

Antibody therapy represents an exciting new treatment approach for childhood leukemia. ADCs and BiTEs are rapidly emerging as the next frontier in the treatment of hematological malignancies and their application in pediatric leukemia is in development. Over the past 50 years, minimal changes have occurred in the drugs used to induce and maintain remission in pediatric leukemia, with most trials using established cytotoxic drugs but with variations in schedules and dosages. Further advancement in the treatment of pediatric leukemia, with the ultimate aim of improved OS and reducing the acute and long-term complications of treatment, may be achieved by the inclusion of novel antibody therapies. Several drug conjugates and bispecific antibodies have demonstrated promising activity in pediatric leukemia and ultimately these compounds may transform the routine management of childhood leukemia patients in the future.

A major challenge lies in the development of clinical trials that will ultimately inform the integration of novel antibody therapies into standard treatment protocols. Experience with rituximab has shown that the improvement in survival in adult lymphoma patients occurs through combination with standard chemotherapy, rather than implementation as monotherapy (16). Trials of antibodies such as alemtuzumab, as single agents in pediatric leukemia, have had difficulty recruiting patients and have shown low levels of activity. Preclinical data on SAR3419 demonstrate that it has the greatest levels of activity and synergy when combined with standard cytotoxic therapies (62). Encouragingly, epratuzumab is the first monoclonal antibody to be evaluated in combination with conventional chemotherapy for childhood pre-B ALL in an international Phase III trial. Integration of antibody therapy with chemotherapy will be especially challenging for BiTE drugs such as blinatumomab that rely on T cell function for their efficacy, since these immune cells are depleted by myelosuppressive chemotherapy. Blinatumomab may eventually have a role administered between cycles of standard chemotherapy, as part of maintenance treatment, post transplantation, or to treat MRD.

The incorporation of antibody therapies into standard chemotherapy backbones not only produces opportunities to increase treatment efficacy, but may be an avenue to reduce treatment side effects. ADC therapies could potentially be used to replace a cytotoxic drug in standard protocols with an ADC with a similar mechanism of action, e.g., vincristine may be replaced in re-induction regimens by SAR3419. Optimal treatment regimens may include a number of ADCs targeting different antigens, e.g., anti-CD20 and anti-CD19 for B cell malignancies. As the number of ADCs developed increase, combination trials will need to be conducted (32).

As antibody trials in pediatric leukemia progress it will be critical to investigate and identify biomarkers that can accurately identify patients most likely to benefit from antibody therapy. While it is clear that expression of the target antigen on the surface of the leukemia cell is a prerequisite, it is unknown whether other factors influence treatment response. For example, are there factors that increase the likelihood of the emergence of a resistant clone, and does the antigen density (that is, the amount of antigen expressed on the surface of each cell) predict response?

Another major clinical challenge in relapsed ALL remains the ability to target sanctuary sites such as the CNS and testicular leukemia. Antibodies in general do not penetrate the blood–brain barrier and preclinical data confirm that novel ADCs do not eliminate CNS disease in mouse models of pediatric ALL (62). Rituximab has recently been shown to be active when administered intrathecally (77, 78). It would be of interest to study the intrathecal administration of novel antibody therapies in childhood leukemia to determine whether this approach may improve the treatment of CNS disease, and also potentially reduce the need for radiation therapy with its associated significant long-term morbidities.

The adverse effects of antibody therapies will need to be closely monitored in the pediatric setting. Overall they appear to be very safe, with the majority of trials showing favorable toxicity profiles. In particular, limited hematological toxicity has been recorded. However, some unexpected adverse events have been noted including neurotoxicity with blinatumomab and an increased risk of VOD, particularly after HSCT, following treatment with GO (4).

It is notable that in the adult population antibody therapy is utilized and studied more often in lymphoma patients than in leukemia patients. This is mostly related to the relative incidence of the diseases, with lymphoma occurring with much higher frequency than ALL. However, hematological malignancies provide ideal targets for these novel agents, as target antigens can be readily assessed by flow cytometry on blood or marrow. In pediatric patients, relapsed ALL is a more common clinical problem with greater burden of disease than lymphoma and should be the focus of future antibody trials in this population.

Several challenges remain to ensure these novel agents are made available to childhood leukemia patients. The cost of development, production, and manufacturing of these drugs is a major limitation to their generalized applicability (9) and pediatric leukemia patients remain a small market for pharmaceutical companies. Despite being the most common malignancy to affect children, relapsed childhood leukemia remains a relatively rare disease, and testing these drugs in clinical trials – from early to advanced phases – requires multi-institutional trials and international co-operation. Despite these challenges, these novel agents bring the promise of great advancements in the treatment of pediatric leukemia with the potential for improved OS and a reduction in treatment toxicity.

## Conflict of Interest Statement

The authors declare that the research was conducted in the absence of any commercial or financial relationships that could be construed as a potential conflict of interest.
